# In silico homology modeling of dengue virus non-structural 4B (NS4B) protein and its molecular docking studies using triterpenoids

**DOI:** 10.1186/s12879-024-09578-5

**Published:** 2024-07-10

**Authors:** Sajid Ali, Usman Ali, Khushboo Safi, Falak Naz, Muhammad Ishtiaq Jan, Zafar Iqbal, Tahir Ali, Riaz Ullah, Ahmed Bari

**Affiliations:** 1 Department of Chemistry, Bacha Khan University, Charsadda, Khyber Pakhtunkhwa Pakistan; 2 Department of Chemistry, Kohat University of Science and Technology, Kohat, 26000 Khyber Pakhtunkhwa Pakistan; 3 College of Medicine, King Saud University, P.O.Box 7805, Riyadh, 11472 Kingdom of Saudi Arabia; 4 State Key Laboratory of Chemical Oncogenomics, Guangdong Provincial Key Laboratory of Chemical Genomics, Peking University Shenzhen Graduate School, Shenzhen, 518055 Guangdong PR China; 5 Department of Pharmacognosy, College of Pharmacy, King Saud University, Riyadh, 11451 Kingdom of Saudi Arabia; 6 Department of Pharmaceutical Chemistry, College of Pharmacy, King Saud University, Riyadh, 11451 Kingdom of Saudi Arabia

**Keywords:** Dengue virus, NS4B protein, Molecular docking, Triterpenoids

## Abstract

**Background:**

Dengue fever has become a significant worldwide health concern, because of its high morbidity rate and the potential for an increase in mortality rates due to lack of adequate treatment. There is an immediate need for the development of effective medication for dengue fever.

**Methods:**

Homology modeling of dengue virus (DENV) non-structural 4B (NS4B) protein was performed by SWISS-MODEL to predict the 3D structure of the protein. Structure validation was conducted using PROSA, PROCHECK, Ramachandran plot, and VERIFY-3D. MOE software was used to find out the in-Silico inhibitory potential of the five triterpenoids against the DENV-NS4B protein.

**Results:**

The SWISS-MODEL was employed to predict the three-dimensional protein structure of the NS4B protein. Through molecular docking, it was found that the chosen triterpenoid NS4B protein had a high binding affinity interaction. It was observed that the NS4B protein binding energy for 15-oxoursolic acid, betulinic acid, ursolic acid, lupeol, and 3-o-acetylursolic acid were − 7.18, − 7.02, − 5.71, − 6.67 and − 8.00 kcal/mol, respectively.

**Conclusions:**

NS4B protein could be a promising target which showed good interaction with tested triterpenoids which can be developed as a potential antiviral drug for controlling dengue virus pathogenesis by inhibiting viral replication. However, further investigations are necessary to validate and confirm their efficacy.

## Introduction

Dengue, a mosquito-borne illness, spreads through the bites of Aedes mosquitoes, specifically such as Aedes albopictus and Aedes aegypti. this disease is widespread across the globe and internationally recognized as the predominant arboviral infection affecting humans [1]. As of the 2023 report, over 3.7 million cases of dengue and more than 2,000 dengue-related deaths have been documented across 70 countries/territories worldwide [2]. An estimated 3.8 billion individuals in 128 countries face heightened risk of infection from dengue viruses (DENVs). Furthermore, approximately 20,000 deaths attributable to dengue occur annually worldwide [3] Dengue disease causes a variety of clinical symptoms, from harmless or normal fever (DF) to potentially lethal hemorrhagic fever (DHF), which is recognized by thrombocytopenia and capillaries leakage. In addition, it can cause shock and circulatory collapse, which is known as dengue shock syndrome (DSS), that is fatal [4]. A single positive-sense RNA strand, comprising the majority of the dengue virus genome, serves as a s for protein synthesis. Initially, this RNA strand translates into a single, long polypeptide chain, which subsequently cleaves into ten distinct proteins. Three structural proteins—the capsid (C), envelope (E), and membrane (M) are included in this group of ten proteins [5, 6]. The dengue virus possesses a spherical structure with a central nucleocapsid primarily composed of C proteins. The E and M proteins, which control the virus entry to human cell, encapsulated the nucleocapsid. The remaining seven proteins are classified as NS1, NS2A, NS2B, NS3, NS4A, NS4B, and NS5. These are all considered non-structural (NS) proteins. These seven proteins participate in viral assembly and replication within host cells [7].

Non-structural transmembrane proteins NS2A, NS2B, and NS4B are essential for the replication process. It has been determined that these proteins block the interferon- α/β (IFN-α/β) responses [8]. All four dengue serotypes contain a small protein called nonstructural 4B protein (NS4B) that has a 40% structure resemblance with other flaviviruses like tick-borne encephalitis virus (TBEV) and yellow fever virus (YFV). The NS4B protein is produced by a single long cleavage polypeptide of the enzyme signalase and serine at the N- and C-terminus of the protease, respectively (NS2B/NS3) [9].

The dengue virus non-structural protein NS4B (DENV-NS4B) is highly hydrophobic and least targeted for drug development among DENV proteins. It is attached to the membrane of the endoplasmic reticulum on its lumen side. Three transmembrane domains (TMDs), designated TMD-3, TMD-4, and TMD-5, are present in the protein’s hydrophobic N-terminal region, which occupies residues 1–93. TMD3 penetrates the endoplasmic reticulum’s lumen membrane to reach the cytoplasmic side, whereas TMD-4 builds a barrier between the cytoplasm and the lumen side. TMD-5 facilitates C-terminal cleavage by spanning the membrane from the cytoplasmic side and then returning to the lumen of the endoplasmic reticulum. It effectively block the STAT1 phosphorylation [10]. During viral replication and anti-host reaction, NS4A and NS4B work collectively present in all dengue viruses and crucial for membrane arrangement that leads to formation of viral replication complex [11].

Currently there is no specific antiviral medication available. However, analgesics, fluid replacement, and bed rest are supportive treatment [12]. There is no available drug that has been proven to be effective against dengue disease. On the other hand, acetaminophen can be used to treat fever and reduce other symptoms. Avoid using other medication such as aspirin, NSAIDs, and corticosteroids [13]. Potent anti-dengue drugs are still necessary despite extensive research efforts. Although few clinical trials to evaluate repurposed drugs such as chloroquine, prednisolone, balapiravir, celgosivir, and lovastatin that have not been able to reduce viral load and antigenemia or have any positive effects on dengue patients [14]. Thus, it is essential to provide precise category of effective medications to treat DENV infection [15].

Medicinal plants provide a rich source of chemical compounds for drug discovery, comprising diverse phytochemicals effective against a range of diseases and infections [16].

Triterpenoids, comprising six isoprene (C5H8) units with a 2-methyl-1,3-butadiene group, possess significant pharmacological and biological activities, particularly against viruses, tumors, and inflammation [17]. Recent literature has increasingly emphasized the remarkable antiviral properties of triterpenoids, which can inhibit the initial stages of viral adsorption and invasion into host cells, thereby blocking viral replication [18].

In the present study, we design the *in silico* homology model of DENV NS4B protein and molecular docking to determine the potential inhibitory effect of five triterpenoids, including 15-oxoursolic acid, betulinic acid, ursolic acid, lupeol and 3-o-actylursolic acid, against the DENV NS4B protein. The homology modeling was performed using the SWISS-MODELL to construct the 3D structure of DENV NS4B protein. However, after validating the target protein structure, the docking studies of selected ligands were performed using MOE 2015.10 software. The main objective of the present study is to treat dengue virus infection by targeting and inhibiting Dengue virus non-structural 4B protein (DENV NS4B) using different triterpenes, amongst which 15- oxoursolic acid is a newly isolated pentacyclic triterpene, as potent inhibitor of the said protein. Triterpenes were selected for this study as different types of them exhibited promising biological activities including antiviral potential. This is a new strategy for the prevention of the hazardous caused by dengue virus infection by inhibiting DENV NS4B.

## Materials and methods

### Homology modelling

As the experimental crystal structure of the DENV NS4B protein is not contained in the Protein Data Bank (PDB), a 3D model of the protein was created. From the UniProt Knowledgebase (UniProtKB), the target protein ID (Dengue virus type 2) was obtained with the accession number P29990. The protein ID was then transmitted to the SWISS-MODEL web service in order to generate a model with sufficient query sequence coverage and sequence identity. The most accurate 3D structure has been selected after considering the values of the Global Model Quality Estimation (GMQE) and Qualitative Model Energy Analysis (QMEAN). The GMQE (Global Model Quality Estimation) values typically range from 0 to 1, with increasing values indicating greater confidence in the predicted molecular structure. Conversely, for QMEAN (Qualitative Model Energy Analysis), values below 4.0 indicate greater reliability in the predicted structure [19].

### Secondary structure analysis, model refinement, and validation

The Phi/Psi Ramachandran plot from the PROCHECK analysis was examined in order to validate the models produced by SWISS-MODEL. The SWISS-MODEL model was finally selected for additional research based on its geometry, 3D alignment with the template, and the outcomes of PROCHECK and PROSA software. The PROSA software was used to compare the predicted models’ energy parameters to currently available X-ray and NMR structures. To verify the interaction energies of each residue in the projected model, the PROSA II energy plot was generated. Furthermore, VERIFY-3D assessed the model’s quality. The VERIFY-3D compares a model’s three-dimensional and sequence score (3D-1D) with its template structure. PROCHECK analysis was used to produce the Ramachandran plot. Using the PDBeFOLD server and BIOVIA Discovery Studio, the projected model was further compared to a template structure (PDB ID: 5zkq.1.A) [20].

### Ligand selection and preparation

The five triterpenoids 15-oxoursolic acid, betulinic acid, ursolic acid, lupeol and 3-o-actylursolic acid were used as a ligand. The stem bark of the *Rhododendron arboreum* was used to isolate the chemical compounds using column chromatography [21]. The ligand molecules chemical structures were drawn by ChemBioDraw Ultra, saved in .*mol* format and subjected to Molecular Operating Environment (MOE) for 3D protonation [22].

### Refinement of receptor protein

The Dengue virus NS4B protein three-dimensional (3D) structure was refined by removing water molecules, energy minimization, and 3D protonation using the Molecular Operating Environment (MOE). Additionally, energy minimization was carried out using the following parameters: (Chiral Constraint: Current Geometry, gradient: 0.05, and Force Field: AMBER12EHT). In order to conduct docking analysis, the generated structure was used as the receptor [23].

### Molecular docking

Using the MOE 2015.10 program, the docking procedure was carried out. The ‘Dock’ function of the ‘Compute’ panel was initially selected to start the docking simulation procedure. Then, keeping a 30% retention value, we chose the “Triangle Matcher” positioning strategy and used LondondG’s rescore (1) feature. In addition, the “Forcefield” refinement technique was selected, and then the Generalised Born solvation model (also known as GBVI/WSA) was used to calculate the scoring function (2) while keeping the value at 1. The settings for the rest of the parameters were left at their default values. The docking outcome can be stored as a file under the.mdb form. Based on their molecular interaction (by looking at the protein-ligand complex from the “LigX” feature) and the Gibbs free binding energy that the simulation generated, the best ligand was chosen through docking simulation [24].

## Results

### Homology modeling of DENV NS4B protein

SWISS-MODEL was used to generate a 3D structure of the DENV NS4B protein with a GMQE (Global Model Quality Estimation) of 0.13 and a QMEAN (Qualitative Model Energy Analysis) of approximately 0.94. Additionally, it was discovered that the DENV NS5 protein (PDB ID: 5ZQK.1; resolution: 2.30 Å) had a comparable template to DENV NS4B, with a similarity identity of 97.33% and a sequence similarity of 0.62. The modeled structure is accurate and of good quality, as evidenced by the values of the GMQE (0.13) and QMEAN (0.94).

**Fig. 1:**
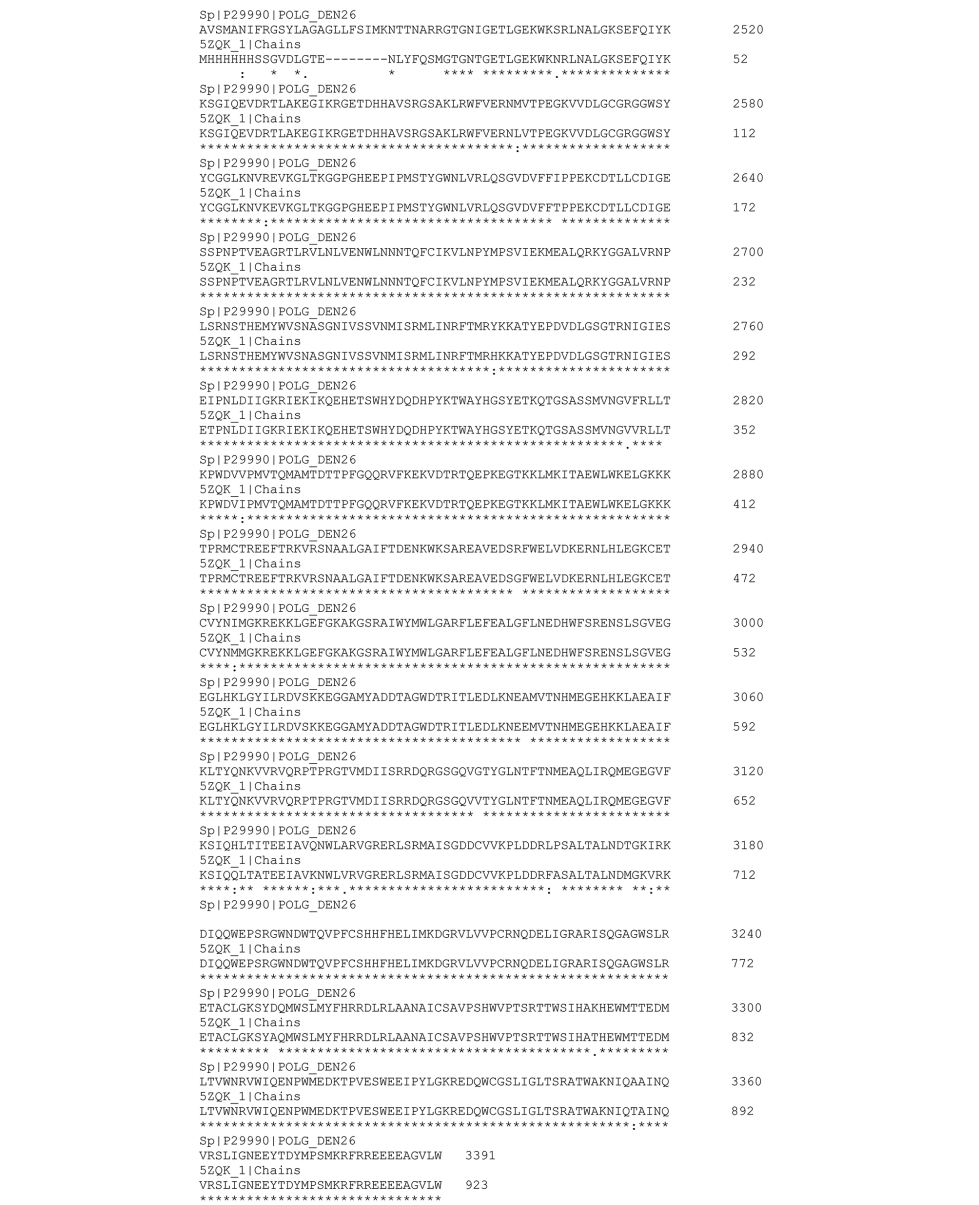
Alignment of the DENV NS4B protein amino acid sequences with the Dengue Virus Non-Structural Protein 5 (5ZQK.1) crystal structure

The amino acid sequences for DENV NS4B (UniProtKB ID: P29990) and NS5 (PDB ID: 5ZQK.1) are multiple sequences aligned in Fig. 1. The similarity identity of 97.33% that was achieved through the method of homology modeling was confirmed by a percentage identity matrix of 95.23%.

### Secondary structure analysis, model refinement and validation

**Fig. 2:**
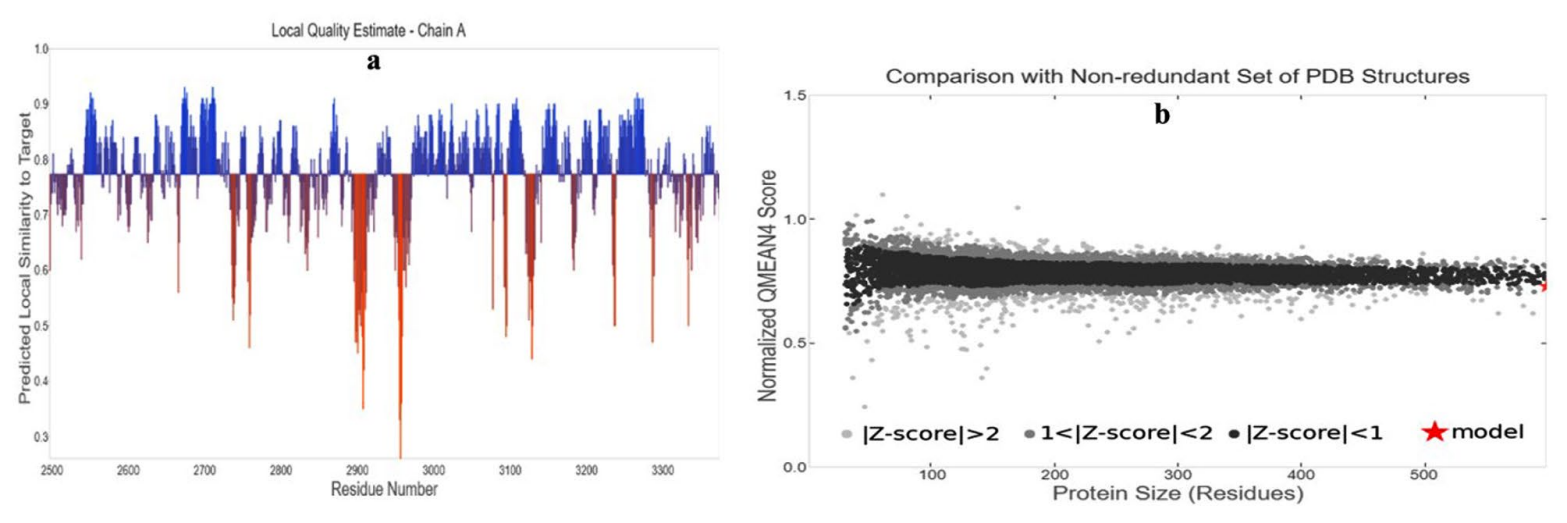
Structure validation of the modeled dengue virus (DENV) non-structural 4B (NS4B) protein (**a**) Local quality estimation of the predicted dengue virus non-structural 4B (NS4B) protein residues (**b**) Comparison of the predicted dengue virus non-structural 4B (NS4B) protein structure with a non-redundant set of PDB structures

**Fig. 3:**
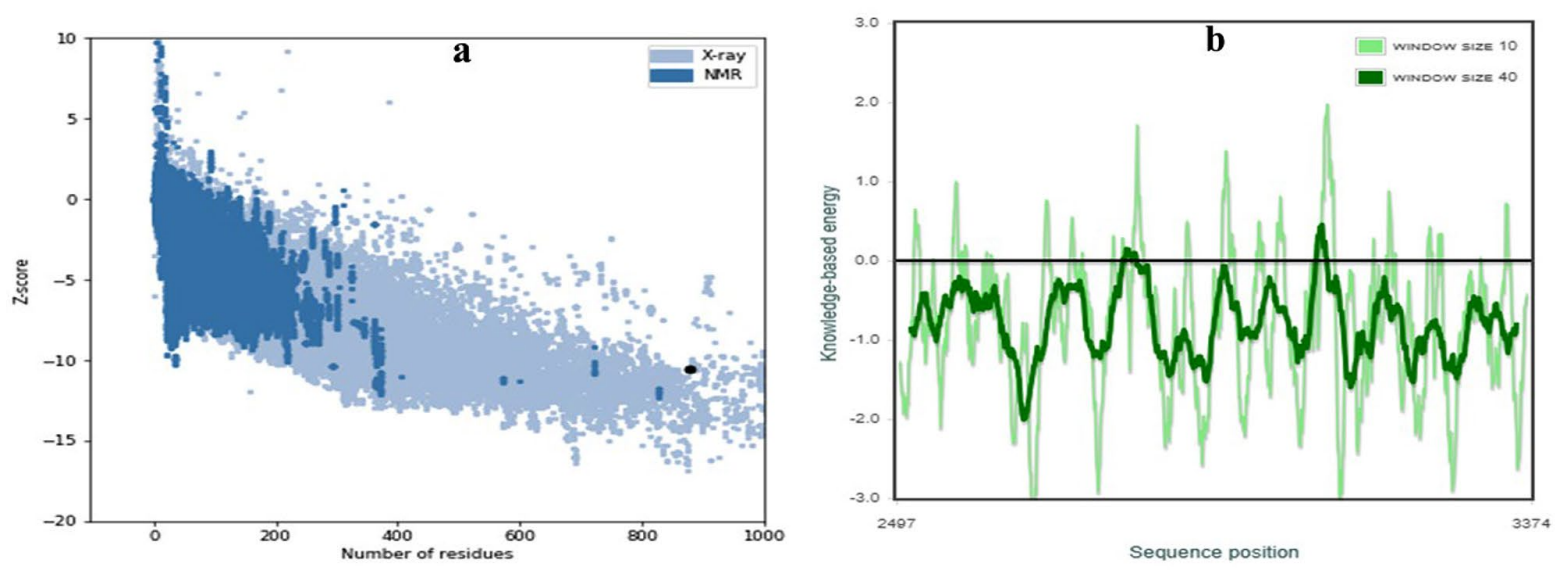
(**a**)The black dot clarifies the similarity of model with X-ray and NMR structures (**b**) Using ProSA, the dengue virus (DENV) non-structural 4B (NS4B) protein model is validated

The projected local similarity to the target, as a function of the predicted 3D structure of the modeled protein’s residue number is shown graphically in (Fig. 2a). The majority of the residues had values close to 1, demonstrating a high level of confidence in the local quality evaluation of the residues predicted by the model. Residues with values under 0.6 were thought of as having low quality. The alignment of the protein structure model with other protein structures in the PDB, as shown in Fig. 2b, provides evidence of its accuracy. Additionally, the PROSA tool was chosen to evaluate the model’s quality by the Z score of the structure. The target structure is examined to see if it falls within the Z score range that is generally observed in proteins of a similar size. The Z score is a measure of the overall model quality. The DENV NS4B protein model’s Z score was − 10.6. The DENV NS4B protein structure was within the range that was acceptable by X-ray and NMR studies, which were in accordance with the PROSA tool’s results (Fig. 3a). Only a small number of residues showed positive interaction energy according to the PROSA analysis of the model, while the majority of residues had negative interaction energy (Fig. 3b).

**Fig. 4:**
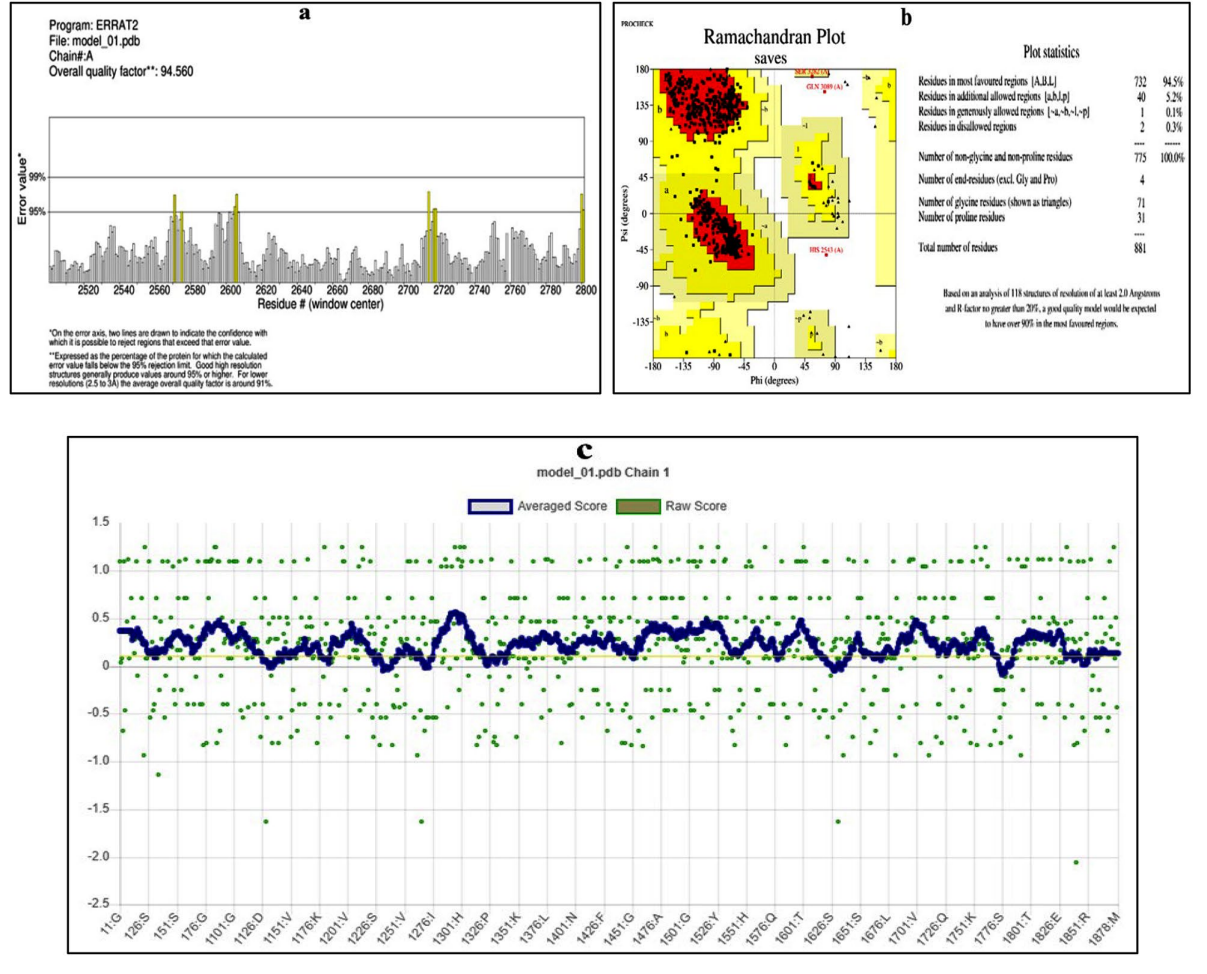
Structure validation using (**a**) the overall quality factor (**b**) the Ramachandran plot of the dengue virus (DENV) non-structural 4B (NS4B) protein homology model, and (**c**) the Verify3D plot

The Procheck and Errat programs provided the Overall Quality Factor (Fig. 4a) and Ramachandran plot (Fig. 4b). A result higher than 90 is often acceptable for ERRAT, which measures the interactions between several molecules. The score of ERRAT in our model was discovered to be 94.56%, which is determined to be a satisfactory value for a typical protein model. The Ramachandran plot statistics show that the DENV NS4B protein’s modeled 3D structure includes 94.5% of its residues in the regions that are most preferred, 5.2% in additional allowed regions, 0.1% in generously allowed regions, and 0.3% in banned regions. This result confirms the validity and applicability of the 3D structure model that was created. Additionally, the protein model was subject to structure validation using a Verify3D plot (Fig. 4c) and demonstrated a successful outcome. According to the 3D environment profile, approximately 80% of the residues exhibit an average 3D-1D score of > 0.1, indicating that the protein model is likely accurate.

### Ligand selection and preparation

**Fig. 5:**
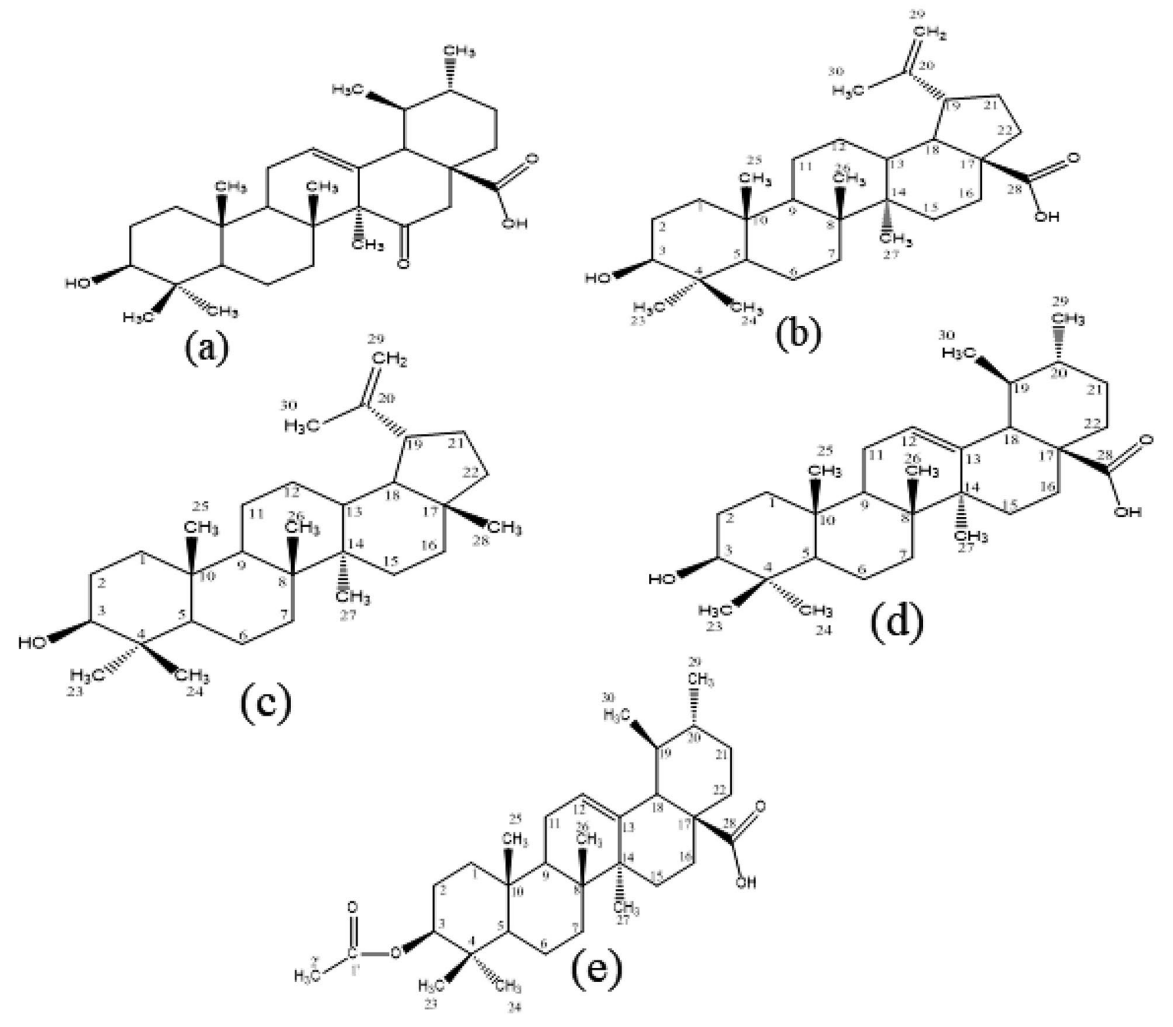
2D Chemical structures of isolated triterpenoids (**a**) 15-oxoursolic acid, (**b**) Betulinic acid (**c**) Ursolic acid, (**d**) Lupeol and (**e**) 3-o-acetylursolic acid

In the present study, five triterpenoids, namely 15-oxoursolic acid, betulinic acid, ursolic acid, lupeol, and 3-o-acetylursolic acid isolated from the stem bark of the *Rhododendron arboreum*, were chosen as ligands to function as a potential inhibitor of DENV-NS4B. The ligand selection and preparation facilitated subsequent stages of the study, thus promoting the evaluation of the ligand’s binding affinities and their interactions with the target. Using energy minimization strategies, the ligands were effectively generated for further analysis and assessment, as shown in Fig. 5.

### Refinement of receptor protein

The SWISS-MODEL 3D structure of DENV NS4B protein was refined using MOE 2015.10. In this process, the water molecules and zinc ions were removed and finally the geometry optimization and energy minimization of the three-dimensional structure of NS4B protein was performed. The Protonate 3D option in MOE was used to repair the structure of NS4B protein and the energy minimization was carried out with the settings (Force Field: AMBER12EHT, Gradient: 0.05, and Chiral Constraint: Current Geometry) was used for docking studies.

### Molecular docking

**Fig. 6:**
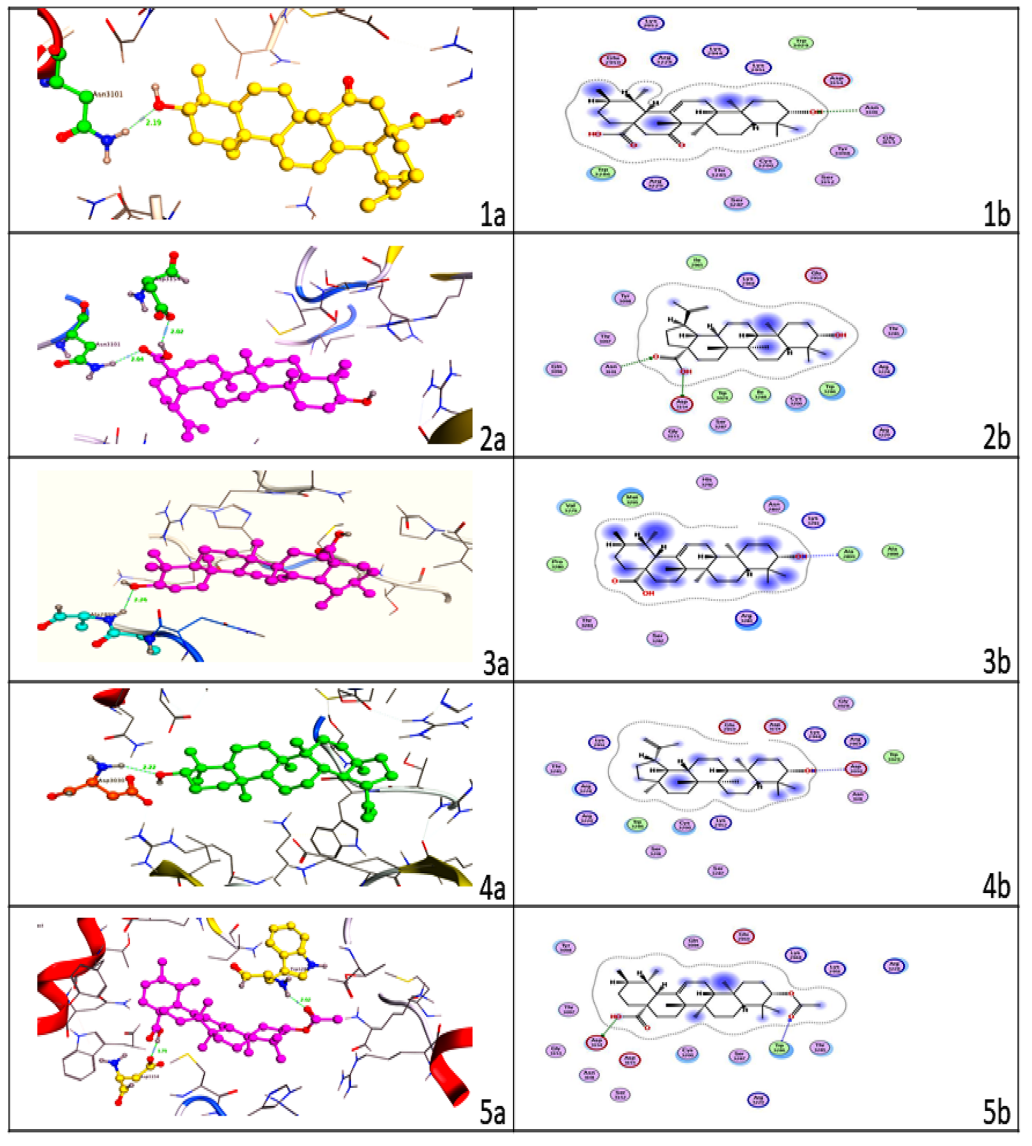
3D and 2D interaction images of triterpenoids, respectively (**1a**, **1b**) 15-oxoursolic acid, (**2a**, 2**b**) Betulinic acid, (**3a**, **3b**) Ursolic acid, (**4a**, **4b**) Lupeol and (**5a**, **5b**) 3-o-acetylursolic acid show interaction with DENV NS4B protein

Docking small molecules into therapeutic proteins is a common technique in drug discovery. *In silico* molecular docking investigations provide a clear description of the drug molecular interactions, whereas small molecule inhibition in cell line tests not fully reveal the ligand-target protein binding orientation. Therefore, a docking study was carried out to identify the amino acid residues of DENV NS4B involved in interaction with triterpenoids and to predict the inhibitory capability of chosen triterpenoids against DENV NS4B protein. Five different types of triterpenoids, including 15-oxoursolic acid, betulinic acid, ursolic acid, lupeol and 3-o-actylursolic acid were selected to study their effect on DENV NS4B protein as shown in Fig. 6. It is clear from the figures that the selected triterpenoids showed good interaction with the amino acid residues in the binding pocket. 15-oxoursolic acid, ursolic acid and lupeol showed interaction with Asn3101, Ala2899 and Asp3030 of DENV NS4B protein with bonding distances of 2.19Å, 2.26Å and 2.22Å, respectively. Similarly, betulinic acid and 3-o-actylursolic acid displayed interaction with Asn3101, Asp3154 and Trp3286, Asp3145 with bonding distances of 2.47Å, 3.20Å and 2.02Å, 1.70Å, respectively. The binding energies for 15-oxoursolic acid, betulinic acid, ursolic acid, lupeol, and 3-o-actylursolic acid were found to be − 7.18, − 7.02, − 5.71, − 6.67 and − 8.00 kcal/mol, respectively (Table 1). The molecular docking results revealed that the selected compounds have good binding affinities with DENV NS4B protein. This indicates that the ligand molecules exhibit a greater affinity for the protein active site and therefore hold a promising place as effective inhibitors for DENV NS4B protein.

**Table 1:** Ligand interaction report of dengue virus (DENV) non-structural 4B (NS4B) protein with triterpenoids

Ligand	Structural formula	Receptor	Distance	E(kcal/mol)
15-oxoursolic acid	C_30_H_46_O_4_	ASN3101	2.19 Å	-7.18
Betulinic acid	C_30_H_48_O_3_	Asn3101, Asp3154	2.47 Å, 3.20 Å	–7.02
Ursolic acid	C_30_H_48_O_3_	Ala2899	2.26 Å	–5.71
Lupeol	C_30_H_50_O	Asp3030	2.22 Å	–6.67
3-o-actylursolic acid	C_32_H_50_O_4_	Trp3286, Asp3145	2.02 Å and 1.70 Å	–8.00

## Discussions

Dengue infection has become a major medical issue. According to the 2023 report, it has affected over 3.7 million people and caused more than 2,000 deaths across 70 countries/territories [1]. About 3.8 billion individuals in 128 countries are at risk of DENV infection, with approximately 20,000 deaths annually reported due to dengue worldwide [2, 3]. The increase in cases of DENV is an important medical problem for which no specific vaccine or effective drug is commercially available [25]. Efforts have been made to develop inhibitors against the NS2B-NS3 protease, NS3 helicase and NS5, but no inhibitors were available for clinical studies [26]. Several reviews have summarized the challenges of developing inhibitors against these enzyme-active viral proteins [27]. These small non-structural membrane proteins represent a new class of targets for antiviral drug development [28]. The strong hydrophobicity of these non-structural proteins of DENV makes possible for them to attach to tiny molecules which are suitable for the development of peptide inhibitors. The discovery of NS4B protein motivates researchers to investigate molecules that can target and inhibit this specific protein without enzymatic activity [29]. The DENV NS4B protein is highly hydrophobic, attached to the lumen side of endoplasmic reticulum and is efficient in inhibition of STAT1 phosphorylation [10]. NS4A and NS4B works together during viral replication and the anti-host reaction which is presents in all dengue viruses, and is crucial for the membrane arrangement that leads to the formation of the viral replication complex [11, 30]. In recent years, researchers have been able to determine the binding affinity of drugs before synthesizing and analyzing them in the lab. Computational methods have offered molecular understanding of very important viral genes, which has been extremely helpful in the discovery of new inhibitory drugs [6].

In the present study *in silico* homology modeling and molecular docking approaches were used. Since the 3D structure of the DENV NS4B protein has not been reported in the Protein Data Bank (PDB), homology modeling was conducted to determine its three-dimensional structure. The structure of the target protein was validated by using ProSA-web validation, Procheck analysis, Errat analysis and Verify3D results. After validation and refinement of the 3D structure of DENV NS4B protein was subjected to molecular docking. The parameters for docking to obtain the accurate docking results were selected carefully to obtained the accurate results. The five triterpenoids 15-oxoursolic acid, betulinic acid, ursolic acid, lupeol and 3-o-actylursolic acid are selected as ligands. It has been reported that different types of triterpenes have outstanding antiviral efficacy [18, 25]. The present study showed that the selected triterpenoids displayed good interaction with the amino acid residues at the active site as indicated by their binding energies. 15-oxoursolic acid, ursolic acid and lupeol showed interaction with Asn3101, Ala2899 and Asp3030 of DENV NS4B protein with bonding distances of 2.19Å, 2.26Å and 2.22Å, respectively. Similarly, betulinic acid and 3-o-actylursolic acid displayed interaction with Asn3101, Asp3154 and Trp3286, Asp3145 with bonding distances of 2.47Å, 3.20Å and 2.02Å, 1.70Å, respectively. The binding energies for 15-oxoursolic acid, betulinic acid, ursolic acid, lupeol, and 3-o-actylursolic acid were found to be − 7.18, − 7.02, − 5.71, − 6.67 and − 8.00 kcal/mol, respectively. Thus, our study suggested that the selected triterpenoids specifically inhibited DENV NS4B protein and blocked viral RNA replication inside the host so could be used as potential candidates for the control of dengue virus infection. However, further studies on test compounds are needed to be used as a drug for the prevention of pathogenesis caused by the dengue virus.

## Conclusion

Since the beginning of human civilization, we have been battling against numerous fatal diseases, and natural products have distinguished themselves as the most effective method of medical therapy for a variety of disorders. The present study aims to investigate natural products, specifically isolated five triterpenoids (15-oxoursolic acid, betulinic acid, ursolic acid, lupeol, and 3-o-acetylursolic acid) from *Rhododendron arboreum*, as potential inhibitors against the NS4B protein of the dengue virus. Through in-silico homology modeling and molecular docking, we have demonstrated their broad-spectrum blocking capacity against DENV NS4B protein. These compounds are the best inhibitors, with value of binding energy of (–7.18, − 7.02, − 5.71, − 6.67 and − 8.00 kcal/mol), binding with important amino acids of the DENV NS4B protein. Further validation through in-vitro and in-vivo studies for designing new potential drugs and highlighting their potential efficacy and to confirm their safety is necessary. This study is very important, as NS4B protein is the target used for the first time for combating dengue infection as till now there is no efficient medicine available to fight against dengue disease.

## Data Availability

All the available data incorporated in the MS and can be found with Sajid Ali.
